# Hemimaxillary Enlargement, Asymmetry of the Face, Tooth Abnormalities, and Skin Findings (HATS) Syndrome: A Case Report and Review of the Literature

**DOI:** 10.7759/cureus.8159

**Published:** 2020-05-16

**Authors:** Abdullah Alakeel

**Affiliations:** 1 Dermatology, College of Medicine, King Saud University, Riyadh, SAU

**Keywords:** hats syndrome, segmental odontomaxillary dysplasia (sod)

## Abstract

Hemimaxillary enlargement, asymmetry of the face, tooth abnormalities, and skin findings (HATS) syndrome, a rare developmental disorder, involves the first and second branchial arches and is characterized by hemimaxillary enlargement, abnormal appearance of skin and teeth, and facial asymmetry. It is generally detected at birth or during early childhood and is associated with unilateral abnormalities of the face, including the bones, teeth, gums, and skin. Becker nevus is the most common cutaneous manifestation of HATS syndrome. Although patients with HATS syndrome have been treated with various therapeutic regimens, no standard or definitive treatment regimen has been established. This study describes this rare condition in a 12-year-old girl.

## Introduction

Hereditary alpha tryptasemia (HαT) is a genetic trait characterized by higher than normal levels of baseline serum α-tryptase resulting from an increased number of copies of the TPSAB1 gene, which encodes α-tryptase [1]. Individuals with higher than normal levels of baseline serum α-tryptase (i.e., 8-10 ng/ml) present with a multi-system disorder called hemimaxillary enlargement, asymmetry of the face, tooth abnormalities, and skin findings (HATS) syndrome, a complex disorder characterized by hemimaxillary enlargement, abnormal appearance of skin and teeth, and facial asymmetry [2,3]. Specifically, the affected portion of the face does not grow as fully as the unaffected portion. HATS syndrome is recognized by the occurrence of segmental odontomaxillary dysplasia (SOD) and hemimaxillofacial dysplasia (HD), along with unilaterally abnormalities of the skin, musculoskeletal system, gums, and teeth [4].

## Case presentation

A 12-year-old girl presented to the dermatology clinic in our institution for the evaluation of an asymptomatic hyperpigmented hairy patch over the right side of the face. This abnormality was present at birth, starting around her right eye and progressively expanding to involve the right side of her face. The patient had not experienced any developmental delays or physical problems, and there was no relevant family history of this disorder.

**Figure 1:**
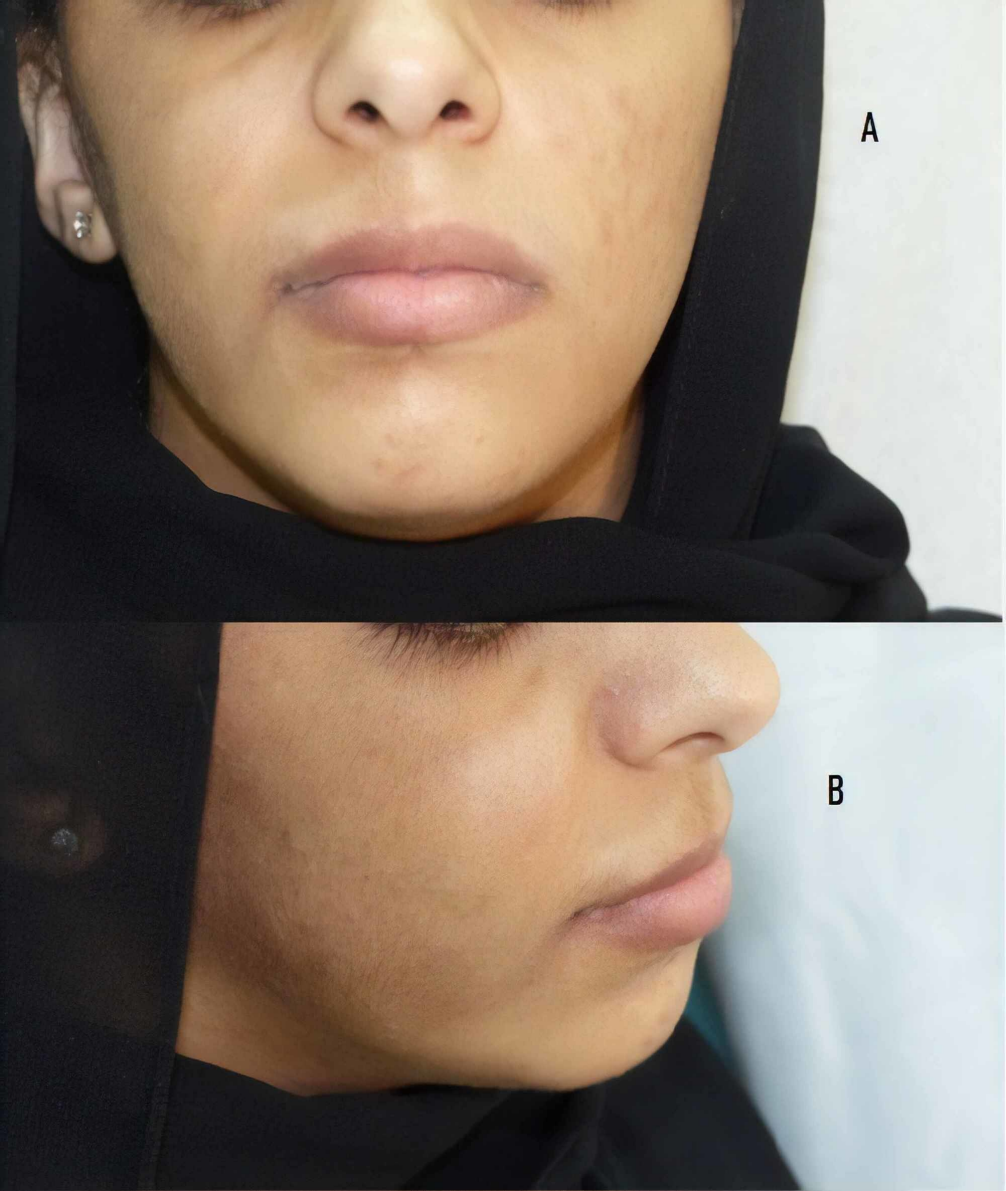
(A) Hyperpigmented hairy patch over right cheek and (B) slight lip hypertrophy over the same side.

**Figure 2:**
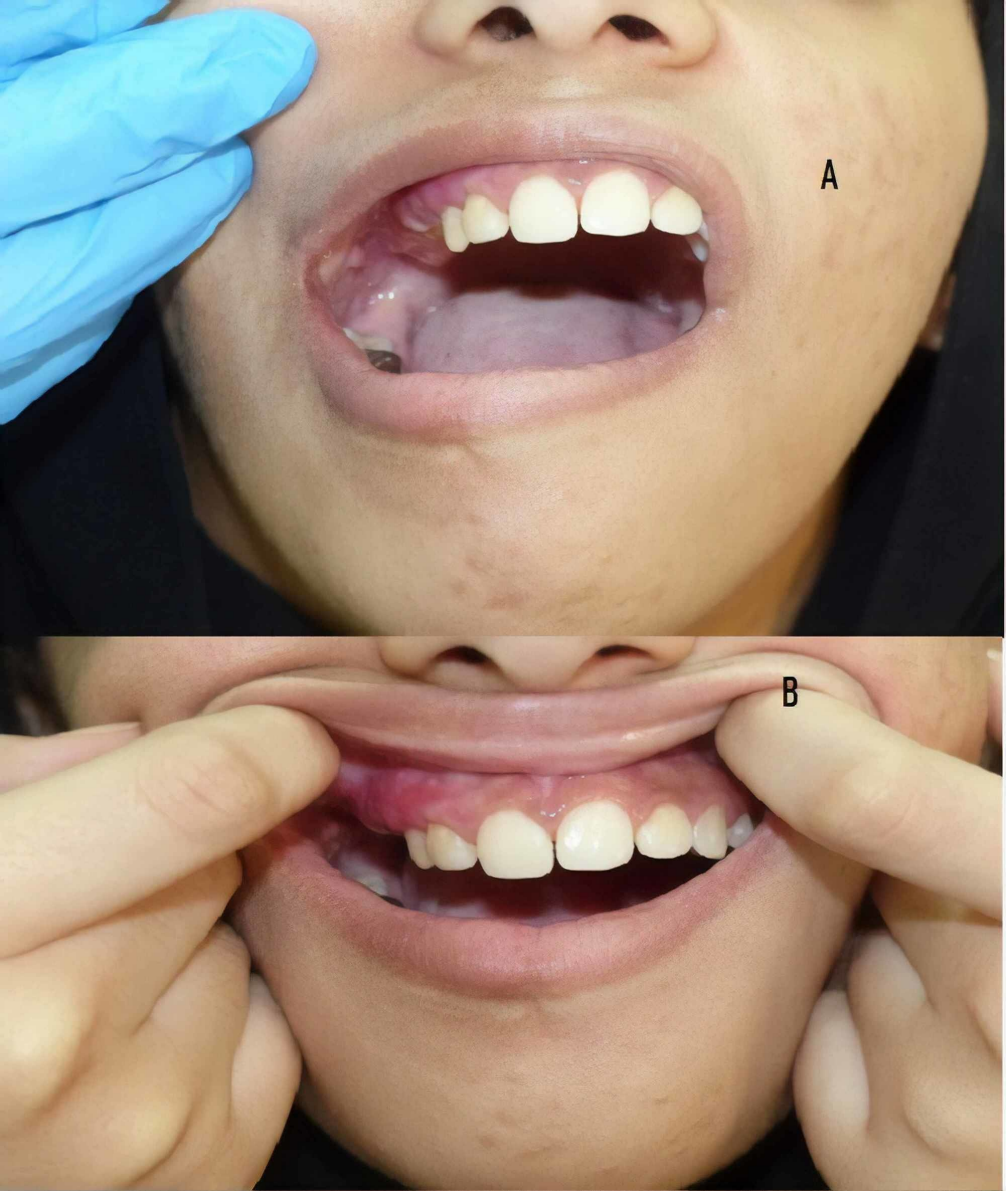
(A) Gingival hypertrophy over the right side and (B) missing and irregular spacing of the teeth on the same side.

**Figure 3:**
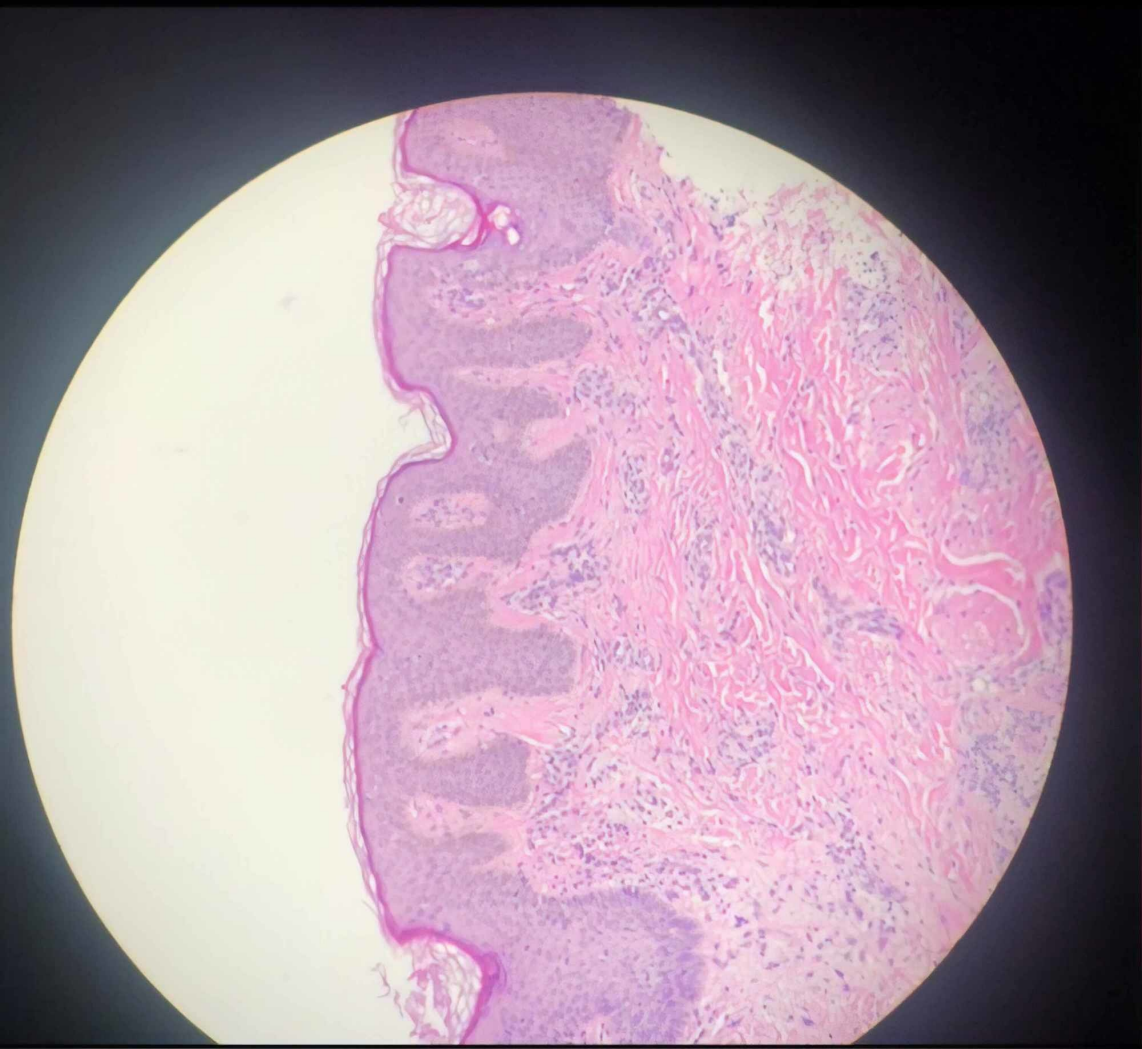
A histopathological appearance of the hyperpigmented patch on H&E staining (high power) and hypomelanosis with elongation of rete ridges, suggestive of Becker’s nevus.

**Figure 4:**
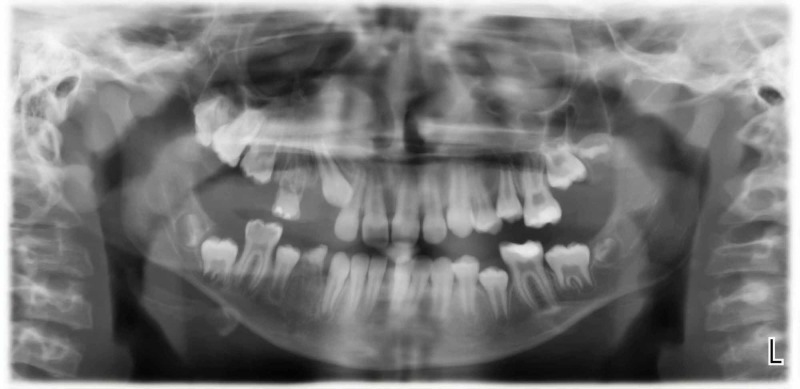
A dental radiograph demonstrating hyperplasia of the right maxillary alveolus and basal bone with missing teeth.

Physical examination showed a well-defined, light brown patch on the right side of her face with hypertrichosis (Figures 1A, 1B), along with slight facial asymmetry. Dental examination revealed a unilateral gingival and lip hypertrophy over the right side, along with irregularly sized spaces between her teeth on her right side (Figures 2A, 2B). Ophthalmologic examination showed that the opening of her right eye was greater than the opening of her left eye, resulting in a diagnosis of hyperopia. A biopsy of the hyperpigmented patch on her right cheek showed basal hypomelanosis with elongation of rete ridges, suggestive of Becker’s nevus (Figure 3). Dental panoramic radiography showed hyperplasia of the right maxillary alveolus and basal bone with missing teeth, consisting of the upper right first and second premolars and the lower right first premolar (Figure 4).

## Discussion

HATS syndrome is considered a mental or developmental disorder usually apparent at birth or during early childhood. It is more prevalent in males than in females, with a ratio of 1.8:1.0, particularly between 2 and 28 years of age. HATS syndrome is characterized by abnormal symptoms of teeth, gums, bones, and skin, which peak during the first 10 years of life [5]. An investigation of two patients in 1987 found that one had a congenitally asymmetric face, with enlargement on one side of both maxillary gingiva and alveolar bone, and the other had hypoplastic teeth, with the affected area showing hypertrichosis [5]. The term hemimaxillofacial dysplasia was suggested. Eight additional patients with unilateral maxillary and gingival enlargement, dental abnormalities, and unique radiographic findings were later described as having SOD, as were patients with similar symptoms but without any changes in the skin [3,6].

Hypopigmentation of the affected part of the lip was reported in a seven-year-old girl with SOD [7]. A case series describes 12 patients with SOD, whereas other case reports described radiographic features in patients with HD or SOD [5,8,9,10]. The term HATS syndrome was based on findings in a patient with Becker’s nevus of the skin [11]. Histopathologic findings of the teeth reported in two patients with SOD included fibrous enlargement of the pulps, an irregular pulp-dentin interface displaying many pseudo inclusions, and pulp stones [12].

In one patient with HD, maxillary dysplasia was varied, as it was due to maxillary hypoplasia rather than hyperplasia [13]. One patient with SOD presented with facial hypertrichosis, commissural lip clefting, and hyper linear palms, whereas another patient presented with unilateral ectopic eyelashes [14,15]. A 14-year-old boy with Becker’s nevus presented with abnormal symptoms including an asymmetric face, hemimaxillary enlargement, and abnormal teeth, all constituents of HATS syndrome [16]. Skin manifestations can vary considerably and can include an asymmetric face, erythema, Becker’s nevus or hairy nevus, hypertrichosis, hypopigmentation of the lip on the affected side, defective vermilion border, and depression [10].

The etiology of HATS syndrome remains unknown. The segmental localization of the abnormalities suggests a local developmental defect [3]. The absence of one or both premolars and early manifestations in some subjects indicate that the disturbance probably occurs in utero, at birth, or during early infancy [17]. A mutation in a progenitor cell early in embryogenesis may give rise to a genetically altered clone of cells, which later colonize or influence the morphogenesis of ectodermal and mesodermal tissues in a segment of the head and neck [18]. A similar postzygotic mutation has been observed in McCune-Albright’s syndrome, resulting in genotypic and phenotypic “mosaicism” of a G protein that causes bone and skin abnormalities [19]. This type of postzygotic mutation in patients with HATS syndrome may result in genotypic mosaicism in bone and skin. Alternatively, HATS syndrome may be due to viral or bacterial infection of the maxillary branches of the trigeminal nerve (V2) [19].

Differential radiographic diagnoses include hemifacial hyperplasia, monostotic fibrous dysplasia, and regional odontodysplasia [6]. No standardized treatment modality has yet been established. Treatments to date include combined surgical and orthodontic treatment of unerupted teeth (premolars/canines), prosthodontic treatment, gingivoplasty, recontouring osteotomy for severe facial asymmetry, and reconstructive jaw surgery [6,8]. Becker’s nevi were reported to be successfully treated with the Q-switched ruby laser, erbium:yttrium aluminum garnet laser, and 755-nm alexandrite laser [20].

## Conclusions

There is a need to describe additional patients with HATS syndrome and to establish an appropriate treatment. Further genetic analysis may detect a novel causative gene in such patients.
